# LipocalinPred: a SVM-based method for prediction of lipocalins

**DOI:** 10.1186/1471-2105-10-445

**Published:** 2009-12-24

**Authors:** Jayashree Ramana, Dinesh Gupta

**Affiliations:** 1 Bioinformatics Laboratory, Structural and Computational Biology Group, International Centre for Genetic Engineering and Biotechnology (ICGEB), Aruna Asaf Ali Marg, New Delhi, India

## Abstract

**Background:**

Functional annotation of rapidly amassing nucleotide and protein sequences presents a challenging task for modern bioinformatics. This is particularly true for protein families sharing extremely low sequence identity, as for lipocalins, a family of proteins with varied functions and great diversity at the sequence level, yet conserved structures.

**Results:**

In the present study we propose a SVM based method for identification of lipocalin protein sequences. The SVM models were trained with the input features generated using amino acid, dipeptide and secondary structure compositions as well as PSSM profiles. The model derived using both PSSM and secondary structure emerged as the best model in the study. Apart from achieving a high prediction accuracy (>90% in leave-one-out), lipocalinpred correctly differentiates closely related fatty acid-binding proteins and triabins as non-lipocalins.

**Conclusion:**

The method offers a promising approach as a lipocalin prediction tool, complementing PROSITE, Pfam and homology modelling methods.

## Background

The lipocalins constitute a group of small (160-200 residues, 15-20KD) mostly extracellular proteins which are highly stable, functionally versatile and widely distributed within different biological kingdoms. The lipocalins belong to the calcyin superfamily, along with fatty acid binding proteins (FABPs), avidins, metallo-protease inhibitors and triabins. In contrast to their poor sequence similarity (identity falling below 20% for paralogs), lipocalins share a highly conserved three dimensional structure. The so-called 'lipocalin fold' comprises a stable calyx-shaped eight-stranded *β*-barrel scaffold, flanked by a C-terminal *α*-helix. The space between the two *β*-sheets of the barrel defines an internal apolar binding cavity with high structural plasticity, consisting of four structurally hypervariable peptide loops, mounted on the barrel. These are divided into two groups according to the presence of three structurally conserved regions (SCRs). The core set of lipocalins called kernel lipocalins share the three SCRs and are more closely related. The more divergent, outlier lipocalins, share only one or two SCRs [1].

Though first identified for their ability to transport small hydrophobic molecules like steroids, bilins, fatty acids etc., it is now established that the functional repertoire of lipocalins encompasses regulation of immunological (e.g. human lcn2 confers mucosal immunity in the respiratory tract [2,3]) and developmental processes, enzymatic (endopeptidase) activities, as for uterocalin, metabolic homeostasis [4], etc. The lipocalins are implicated in various environmental stress responses (in plants- e.g. AtTIL and in bacteria- e.g. YodA ), allergies, as candidate markers of kidney function [5], and acute phase response of inflammation [6]. The lipocalins are also known to interact with membranes.

The flexibility of lipocalin ligand binding pocket has invigorated greater interest in lipocalins, with Pieris AG, Germany now developing artificial lipocalins having novel binding specificities, denominated as Anticalins [7,8]. Anticalins offer a far more lucrative technology over the conventional antibodies as promising reagents for research, biotechnology and therapeutic applications.

In the light of ever increasing wealth of genomic data and the burgeoning interest in lipocalins, it becomes important to have facile methods of lipocalin identification. Prediction of few well characterized lipocalin family members is missed by the PROSITE, Pfam signatures (e.g. apolipoprotein M) as well as Position-Specific Iterative-Basic Local Alignment Search Tool (PSI-BLAST) [9] searches (discussed in the results section). Easy and reliable identification of lipocalins remains an arduous task, attributable to extreme sequence diversity amongst lipocalins. Crystallization and structure solution remain the only certain ways of identification of novel lipocalin family members [10] and indeed, a number of crystal structures have been solved for lipocalins. Experimental determination, however requires expensive infrastructure, and is labour- and time-intensive. Computational structure determination methods like homology modelling and threading, offer an easier alternative and have been used as in the case of apolipoprotein M [11,12]. Another recent improvement to such methods for recognizing distantly related members, involves the identification of crucial interactions involving two conserved clusters of hydrophobic residues [13]. But these methods are fraught with complications first in their own right and secondly due to the difficulty in selecting the template for modelling, since the minimum similarity for reliable modelling is 30% [14].

Machine learning techniques present an alternative, reliable and faster solution for such problems. First pioneered by Vapnik in 1995, Support Vector Machine (SVM) is one such supervised learning algorithm which delivers state-of-the-art performance in recognition and discrimination of cryptic patterns in complex datasets [15]. SVMs are used in conjunction with kernel functions which implicitly map input data to high dimensional non-linear feature space. SVMs construct a large margin hyperplane separating the training data in this space with the aim of achieving minimum classification error [16]. Apart from the classical kernels-linear, polynomial, RBF and Gaussian kernels, there exist variety of sequence-specific string kernels [16-18]. SVMs premised on a strong theoretical underpinning [19] have been used extensively across a growing spectrum of applications in computational biology because of their ability to deal with high-dimensional, large and diverse types of datasets as well as to effectively handle noise [20]. SVM-based prediction methods have been successfully employed for a legion of biological problems, including identification of DNA and RNA binding proteins [21], post-translational modifications in proteins [22], automated classification of microarray gene expression profiles [23], etc. In the present study, we developed a SVM-based method to facilitate the prediction of members of lipocalin family. Apart from composition-based features, that is amino acid composition (AAC), dipeptide composition (DPC) and secondary structure composition (SSC) obtained from Protein Structure Prediction (PSIPRED) [24], we used the Position-Specific Scoring Matrix (PSSM) profiles obtained from PSI-BLAST for training the SVM models.

Given the mounting interest and biotechnological applications of lipocalins, we hope this would be a useful tool to the end-user biologist and the research community as a whole.

## Results and Discussion

### Algorithm

#### Performance of similarity-based searches

We carried out PSI-BLAST analysis on the non-redundant positive dataset in a fashion similar to the LOO CV (Leave One Out, Cross Validation), with the default values of E as 0.001 and the number of iterations as 3. Each sequence served as the query sequence once while the remaining formed the database, with the procedure iterating on each sequence. Herein, no significant hits were obtained for 6 sequences; thereby reinforcing that even remote similarity based searches may miss out some of the positive hits i.e. lipocalins.

With the tremendous increase in the number of sequences accumulating from different sequencing projects, the number of such sequences may be substantially high because of the absence of any lipocalin hit in similarity-based searches. This explains the need for methods specific for lipocalin identification to complement such general similarity-based methods of protein annotation. Thus, we embarked upon exploring machine learning methods based on various protein features to facilitate lipocalin identification.

#### Performance of standalone SVM models

We began with the LOO Cross-Validation of AAC, DPC, SSC and PSSM based classifiers, trained using the three kernels-linear, polynomial and RBF (Radial Basis Function). Thereafter, hybrid models using combination of two or more features were also developed. The hold-out procedure was performed for the best classifiers to further assess the discriminative quality of the models. Hold-out method provides a further reinforcement about the discriminative power, though because of the random partitioning of the datasets, the results may vary considerably for the different sets [25]. Table 1 (and the table in additional file 1) summarise the performance of the best SVM classifiers for each module and with each kernel as observed in the cross-validation tests.

**Table 1:** Performance of SVM classifiers for various combinations of training features, kernels, parameters and validation methods

Feature	V*	Kernel	Parameters				SN (%)	SP (%)	Acc (%)	MCC	F measure
			**Threshold**	**C**	***γ***	**d**					

AAC	A	R	-0.1	1	0.01	-	72.79	77.10	75.16	0.498	1.429
DPC	A	P	-0.1	0	-	2	80.14	87.34	84.10	0.678	1.658
PSSM	A	R	-0.1	5	9	-	89.70	89.15	89.40	0.786	1.725
	D	R	-0.1	5	9	-	84.55	85.54	85.09	0.701	1.644
	D	R	-0.1	5	9	-	88.96	84.33	86.42	0.731	1.633
SSC	A	R	-0.1	5	3	-	86.02	86.74	86.42	0.726	1.665
	D	R	-0.1	5	3	-	84.55	86.74	85.75	0.712	1.664
	D	R	-0.1	5	3	-	82.35	78.91	80.46	0.609	1.509
DPC+SSC	A	P	0.1	0	-	2	85.29	86.14	85.76	0.713	1.651
PSSM+SSC	A	R	0.0	4	1	-	88.97	92.16	90.72	0.812	1.785
	A	R	-0.1	4	1	-	89.70	89.15	89.40	0.786	1.725
	D	R	0.0	4	1	-	87.49	80.72	83.77	0.678	1.561
	D	R	0.0	4	1	-	85.29	84.93	85.09	0.700	1.628
DPC+PSSM	A	R	-0.1	0	0.001	-	81.61	83.73	82.78	0.652	1.592
DPC+PSSM+SSC	A	P	0.1	0	-	2	85.29	86.14	85.76	0.713	1.651

*Validation: A = Leave-one-out; D = Hold-out

Kernel: R = RBF; P = Polynomial; L = Linear

##### Composition based SVM classifiers

We obtained accuracies of 69.20 and 74.83% in AAC-based SVM models with the linear and polynomial kernels respectively, and 75.16% with the RBF kernel (Table 1 and the Table in the additional file 1). The accuracies sharply increased with DPC usage and reached 79.80, 84.10 and 82.45% for the three kernels, respectively. However, the SSC model yielded the accuracies of 80.46 and 81.12% respectively with the linear and polynomial kernel whereas with RBF, the accuracy touched a striking high of 86.42% with an MCC of 0.726. The sensitivity and specificity of this model were also appreciably high at 86.02 and 86.74% respectively. The remarkably better performance of SSC vs AAC and DPC models is congruent with the known structural conservation albeit the high sequential heterogeneity of lipocalins.

##### PSSM profile based SVM classifier

PSI-BLAST derived PSSM profiles captures useful information about the residue composition as well as conservation of residues at crucial positions within the protein sequence, because in evolution the amino acid residues with similar physico-chemical properties tend to be highly conserved due to selective pressure. PSSM profiles have been used as SVM input feature for a number of classification problems, e.g. prediction of sub-cellular localization [26], nucleic acid binding proteins [27], etc.

We used the PSSM profile, normalized using the logistic function (See Methods) for developing an SVM module. The PSSM profile-based model yielded maximal accuracies of 85.43 and 87.41% respectively with linear and polynomial kernels respectively, and a remarkably high accuracy of 89.40% with the RBF kernel, with the sensitivity and specificity of 89.70 and 89.15% along with an MCC of 0.786.

#### Performance of hybrid SVM models

With an aim to further enhance the prediction accuracy, we developed and evaluated four hybrid models using different combinations of features (hybrids).

##### DPC and SSC hybrid

This model performed better than the standalone DPC model but did not achieve any improvement over the SSC model, showing the maximum accuracy of 85.76% with the polynomial kernel. With the linear and RBF kernels, the accuracies were 83.77 and 85.43% respectively.

##### DPC and PSSM hybrid

This hybrid model performed as well as DPC but much worse than the PSSM model and showed a dramatic dip to an overall maximal accuracy of 81.12% with the polynomial kernel. This may be attributable to noise produced by the increase in the length of training input to 800 dimensions. With the linear and RBF kernels also, the performance was comparable at 79.80 and 80.46% accuracies.

##### PSSM and SSC hybrid

This was the model with the highest overall accuracy of 90.72%, better than both the PSSM and SSC models, but with a slightly lower sensitivity (88.97%) and higher specificity (92.16%) as that of the PSSM model. The accuracy was 85.76 and 84.43% with both the linear and polynomial kernels. This model achieved the best overall accuracy amongst all the models.

##### DPC, PSSM and SSC hybrid

This model performed reasonably well, with accuracies of 83.44, 85.43 and 85.76% with the polynomial, RBF and linear kernels respectively. Yet this was not an improvement over any of the models based on one or two features and was therefore not considered for any further evaluation.

#### Hold-out procedure

Hold-out procedure was run on the three best SVM models: PSSM, SSC and PSSM-SSC. This procedure simulates the performance of the classifier over a blind test set since only randomly chosen one-half of the data is used for training while the other half is used for testing. Two runs of hold-out method were therefore carried out at the same parameters as obtained from LOO since this would be used for final prediction. The three classifiers achieved around 85% (Table 1) accuracy in both the runs, reflecting the strong discriminative power of the models.

#### ROC plot

**Figure 1:**
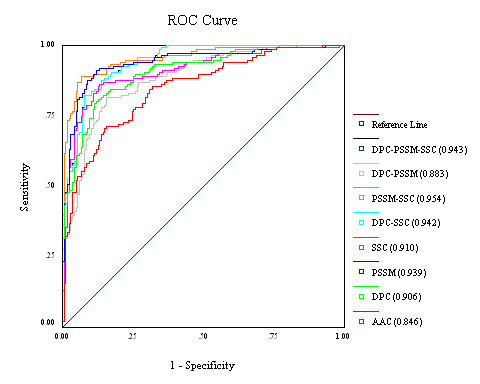
**ROC curves of the different SVM classifiers**. ROC plot of SVMs based on different protein sequence features depicting relative trade-offs between true positive and false positives. The diagonal line (line of no-discrimination) represents a completely random guess. The corresponding area under curve (AUC) is given in brackets in the legends.

ROC curves show the trade-off between true positive rate (sensitivity) and false positive rate (specificity) over their entire range of possible values. It is considered as the most robust approach for classifier evaluation [28]. The area under the ROC curve (AUC) is used as a reliable index of classifier performance. To validate the threshold-independent performance of our SVM models, we compared the ROC curves for the best SVM models obtained for each feature as well as combination of up to three features (Figure 1).

A comparison of AUCs for various ROC curves revealed that the models followed almost the same increasing order of AUC as the prediction accuracies in SVM: AAC (84.6%), PSSM-DPC (88.3%), DPC (90.6%), SSC (91.0%), PSSM (93.9%), DPC-SSC (94.2%), PSSM-DPC-SSC (94.3%), PSSM-SSC (95.4%). Only in one case, namely for the PSSM model, the AUC did not follow the order of prediction accuracy in SVM. There was a complete overlap for the curves for DPC-SSC and PSSM-DPC-SSC as also reflected in their exactly similar accuracies (85.76% for both).

This analysis verified the efficacy of the SVM classifiers. These AUC values are significantly higher than that of random guessing (0.5).

#### Testing

##### Performance on independent datasets

LOO and hold-out tests may give over-optimistic estimates of performances because the model parameters are optimal for the datasets used for training but may not perform well on unseen data [29]. To confirm the behaviour of the models, we tested the performance of the classifiers on independent datasets. Table 2 depicts the performance of the classifiers on three independent datasets- two negative and one positive dataset.

**Table 2:** Quality estimation of SVM models over random prediction

Model	Correctly predicted (total 302)	S (%)
AAC	227	49.869
DPC	254	67.767
PSSM	270	78.653
SSC	261	72.632
DPC-SSC	259	71.297
PSSM-SSC	274	81.247
DPC-PSSM	243	60.772
DPC-PSSM-SSC	259	71.297

The table estimates the quality of the models generated from each module as compared to random prediction(S).

***Negative datasets:*** These consisted of other members of the calcyin superfamily, one set having 25 FABPs and the other with 28 triabins, which are highly likely to be picked up as positives due to sharing evolutionary ties with lipocalins. Due to structural and functional grounds, lipocalins and FABPs have been merged into one Pfam signature (PF00061). However, FABPs have a ten-stranded discontinuous beta-barrel structure as against the continuous eight strand barrel of lipocalins. Triabins also differ from lipocalins in having an inverted stand topology for two beta strands in the beta barrel [1]. Since we aimed to develop a classifier exclusively for lipocalins, we tested the performance of our final models on this set to gauge the selectivity of the models for lipocalins. Whereas the PSSM model could predict all of the 25 FABPs as negatives, only 18 out of 25 triabins were predicted as negatives. The PSSM-SSC and SSC models picked up 25 and 17 FABPS respectively while both predicted all of the 28 triabins as non-lipocalins.

***Positive dataset:*** In order to evaluate the practical prediction ability of the final prediction models, an independent dataset consisting of 42 lipocalin sequences was used. While the PSSM model could predict 39 sequences, the PSSM-SSC model predicted 38 sequences as lipocalins. The SSC model picked up only 34 as lipocalins.

##### Comparison of classifier's performance with random prediction

Table 3 depicts the prediction reliability of all the SVM models using the S measure i.e. the normalized prediction accuracy which compares the prediction efficiency over the random prediction. While the AAC and DPC models stood low with S at 49.87 and 67.76% respectively, the SSC and PSSM models performed fairly better with 72.63 and 78.65% respectively. The DPC-PSSM hybrid was even worse with 60.77 with the DPC-SSC at 71.29%. The triple hybrid DPC-PSSM-SSC model had S as 71.29 whereas the PSSM-SSC performed better than PSSM getting S as 81.247. Thus the three best classifiers were models based on PSSM-SSC, followed by PSSM and SSC.

**Table 3:** Performance on independent datasets

Model tested	Positive (total 42)	Negative-FABPs (total 25)	Negative-Triabins (total 28)
SSC	34	17	28
PSSM	39	25	18
PSSM-SSC	38	21	28

The three best models were used for testing on independent datasets. The numbers show the correctly predicted sequences out of the total given in the first row.

## Implementation

**Figure 2:**
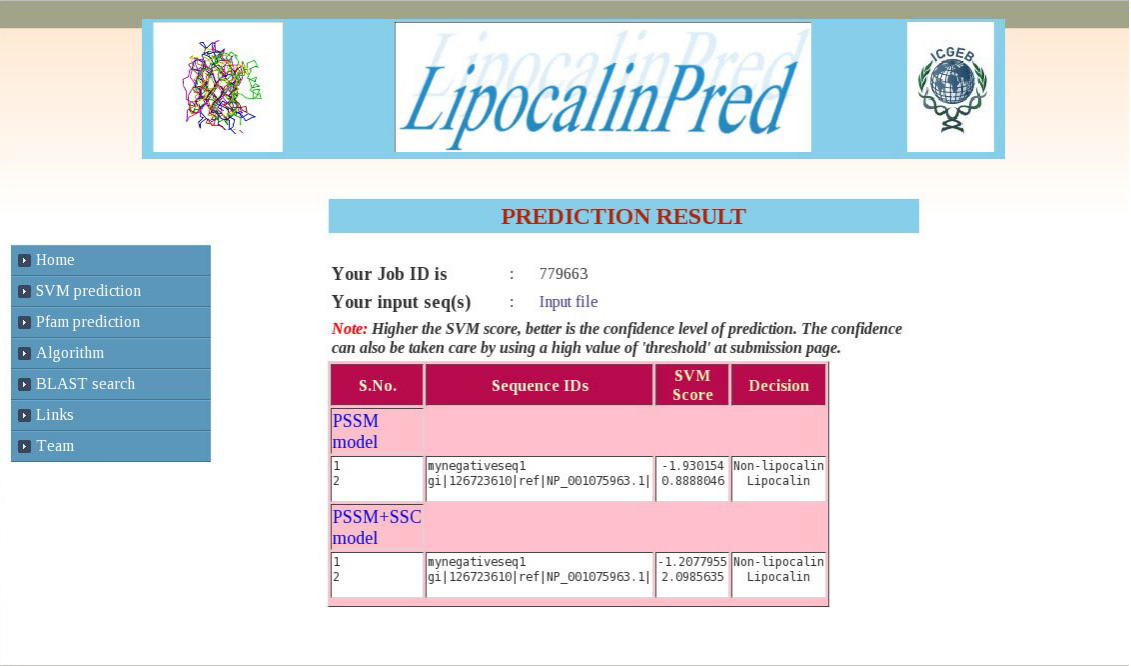
**Snapshot of LipocalinPred web server sample output**. The web server predicts lipocalins based on the two best classifiers, namely based on PSSM profile and the hybrid classifier: PSSM-SSC. The two classifiers may be chosen together for a comparative prediction. The server accepts FASTA formatted sequences and allows user defined thresholds of prediction, ranging from -1.5 to 1.5.

The prediction algorithm presented in this study is implemented as a freely accessible web server at http://bioinfo.icgeb.res.in/lipocalinpred (Figure 2). The web server is hosted on a T1000 SUN server. PHP is used to generate the front-end HTML pages.

The program predicts lipocalins based on the two best classifiers, namely the ones based on PSSM profile alone and PSSM-SSC. It accepts the sequences in FASTA format and allows the user to select different thresholds of prediction ranging from -1.5 to 1.5. The default thresholds on the server are the ones which yielded the best accuracies in cross-validations. The output consists of the sequence ID, the SVM score and the decision of the model regarding the sequence based on the threshold chosen. The higher the SVM score, the better is the confidence level of prediction. The web-server also allows the user to scan a set of sequences for lipocalin-related Pfam signatures. An indexed database of lipocalins is also included to allow the user to search lipocalin sequences sharing regions of similarity to the query sequence set using ViroBLAST [30].

## Perspectives

The performance of AAC and DPC-based models is consistent with the fact that the lipocalins show remarkable diversity at the primary structure level. Hence, it is not surprising that the SVM models trained on AAC and DPC miss out a significant chunk of the training sequences.

For the SSC and its hybrid models, the inability to correctly predict some sequences can be reconciled in light of factors like limitation of the PSIPRED algorithm itself, which is used for secondary structure prediction in the study.

Even the models based on PSSM and its hybrid models fail to predict few lipocalins due to extreme divergence amongst the proteins. The dynamism of evolutionary forces which finally shape the structural and functional aspects of families of diverse proteins like lipocalins results in extreme sequence divergence amongst its members.

The prediction statistics obtained from models based on implementation of features used to develop the best model are highly encouraging. In future, inclusion of additional lipocalin sequences, features and more efficient methods for secondary structure prediction will further improve the efficiency of LipocalinPred.

## Conclusions

Current lipocalin identification methods include experimental determination and homology modelling, which require enormous efforts. The study presented here represents an initiative towards easy identification of lipocalins from other proteins with high selectivity. Apart from solving the lipocalin identification problem in particular, it advocates and reinforces the rational application of machine-learning algorithms like SVMs to classification problems in biology. The study could be extended to other families sharing low pairwise sequence similarity, e.g. to develop an SVM classifier exclusively for FABPs. Since lipocalins are widely spread across the different biological kingdoms, the algorithm may be used for the proteome-wide prediction of lipocalins, especially in cases where their existence is dubious as for archaebacteria. Though identification of a protein sequence as a lipocalin would speak little about function because of the high functional versatility, yet it would provide significant clues about the protein structure and hence lead the way towards providing mechanistic insights about the protein.

## Methods

### Generation of training datasets

A raw pool of lipocalin sequences was compiled from different databases like SwissProt, Refseq and GenBank by keyword search. These were filtered for entries annotated as 'hypothetical', 'putative', 'truncated', 'partial', 'similar to', 'fragment' etc. and then for non-specific hits obtained by keyword search to obtain only full-length and annotated lipocalin protein sequences. The non-lipocalins were also compiled using a similar approach. To remove redundancy in the dataset, the positive and negative datasets were subjected to PISCES [31] program at 40% identity threshold. After this step, the final training datasets consisted of 136 lipocalin and 166 non-lipocalin (Additional files 2 and 3) sequences.

### Datasets for blind test performance

In order to benchmark the unbiased prediction efficiency of our best SVM models, we tested their performance on datasets not used in training or testing. This dataset consisted of 42 lipocalin sequences of which only 3 are more than 60% similar to those in the positive dataset (Additional file 4). 38 out of 42 sequences are no more than 50% similar to positive dataset. We also tested the performance of the models for non-lipocalins comprising closely related family members- FABPs and triabins (Additional file 5).

### Assessment of training dataset for the presence of lipocalin signatures

We tested the positive dataset sequences for the presence of lipocalin related PROSITE (PS00213) and PFAM (PF00061, PF08212, PF02087, PF07137, PF02098) signatures using the locally installed pfscan and HMMER (version 2.3.2) [32] programs respectively. While 88 out of 136 were missed by pfscan, 11 were missed by the five lipocalin-related Pfam signatures and 10 were not picked by Pfam signatures as well as pfscan. This demystifies the fact that all the known annotated lipocalins are not represented by Pfam domains or PROSITE signatures.

### SVM algorithm and problem formulation

We used the freely available package SVM^light ^[33,34] to implement SVM on our training datasets. This package allows optimization of a number of parameters and offers the choice to use different kernel functions to obtain the best classification hyperplane. The separating hyperplane generated by SVM model is given by

Where, *w *is a vector normal to the hyperplane and *b *is a parameter that minimizes  ||*w*||^2 ^and satisfies the following conditions:

for x_i _of one class

for x_i _of the second class.

which may be re-written as:

for all 1 ≤ i ≤ n, n being total number of examples.

However, the above holds true for the linearly separable case which is generally not the case. When the two classes are not linearly separable (e.g., due to noise), the "soft-margin" SVM [35] is used in which the condition for the optimal hyperplane can be relaxed by including a slack variable *ξ*_i_, so that now the optimization problem is to minimise ||*w*||^2 ^+ C*ξ*_i _subject to the following constraint:

for all 1 ≤ i ≤ n.

Here C is a regularization parameter that controls the trade-off between maximization of the margin and minimization of the training error. Small C tends to emphasize the margin while ignoring the outliers in the training data, while large C may tend to over fit the training data.

In the present work, two types of SVM models were developed: (1) based on single sequence features (2) those based on two or three protein features called hybrids.

### Cross-validation methods

We performed training testing cycles using in-house perl scripts. We used linear, polynomial and radial basis function (RBF) kernels to train and test our SVM models. Each kernel was optimized to yield the best classification by changing the kernel parameters (C, d and *γ*). Our approach was to choose the best parameters in a way so as to maximize accuracy as well as get nearly equal sensitivity and specificity, wherever possible.

#### Leave-one-out cross validation (LOO CV)

This is a stringent mode of evaluation wherein one dataset sequence is left out for testing, while the rest are used to generate the model. This is iterated on each sequence till each sequence becomes the testing data exactly once. The best parameters as measured by the various performance measures are picked up and then averaged for the final assessment of the model. It has been shown to give an almost unbiased estimator of the generalisation properties of statistical models, and therefore provides a sensible criterion for model selection and comparison.

#### Hold-out procedure

In this method, the dataset is split randomly into two sets having roughly equal number of training sequences; one is used for training while the other for testing and this is repeated for both sets. The accuracy is then averaged for the two cycles.

### Performance measures

In order to assess the accuracy of prediction methods, we used several measures, namely- Sensitivity: percentage of lipocalin protein sequences that are correctly predicted as lipocalins, Specificity: percentage of non-lipocalin protein sequences that are correctly predicted as non-lipocalins, Accuracy: percentage of correct predictions, for lipocalins as well as non-lipocalins, and Matthews Correlation Coefficient (MCC): a measure of both sensitivity and specificity (MCC = 1 indicates a perfect prediction while MCC = 0 indicates a totally random prediction, F1 statistic: It is the harmonic mean of sensitivity and specificity and is considered a more robust measure as other measures can overstate the performance of the classifier, S: the normalized percentage of correctly predicted samples better than random.

Mathematical representation of the above mentioned measures are defined as follows:

Where, TP is the number of True Positives, TN is the number of True Negatives, FN is the number of False Negatives, and FP is the number of False Positives for a prediction method. Also, *R *is the anticipated number of proteins that are correctly classified by random prediction.

### Protein features and vector encoding

#### Amino acid composition (AAC)

It is the fraction of each of the 20 amino acids present in a protein sequence. This generates a training input vector of 20 dimensions.

where *i *can be any of the amino acids.

#### Dipeptide composition (DPC)

It is the occurrence of a dipeptide divided by the total number of possible dipeptides in the protein which equals one less than the length of the protein. This yields a training input vector of 400 dimensions.

where *j *can be any of the 400 dipeptides.

#### Secondary structure composition (SSC)

High structural propinquity is the hallmark of lipocalin family. Secondary structure prediction was carried out using PSIPRED v2.2.6. It predicts secondary structure for each residue and provides a confidence score for three types of secondary structures: helices, *β*-sheets and coils. The scores for each secondary structure corresponding to a particular residue were added up and divided by residue frequency generating a 20 × 3 matrix, which was used as an input for SVM. This was normalized using the following logistic function:

where x is the raw value in PSSM profile and *g(x) *is the normalized value of x. Following equation was used to calculate the features corresponding to secondary structure prediction,

where *SS *is the score for any of the three secondary structures (helix/sheet/coil) with the summation running over the protein length for each amino acid j. For each j, there exist three F_ss,j _corresponding to each secondary structure. F_i _(j) is the frequency of the amino acid j in the protein.

#### PSSM profile

This was obtained by performing PSI-BLAST against Swissprot (release 57.3) database at the default E-value of 0.001 with three iterations. The matrix contains 20 × N elements, N being the length of the query sequence, and each element represents the frequency of a particular residue substitution at a specific position in the alignment. This was also normalized using the logistic function in the same way as done by us previously in VirulentPred [36]. Following this, the normalized matrix is organized into a composition matrix of fixed length pattern of 400 (20 × 20, for each amino acid, there are 20 substitution scores from normalized matrix).

### ROC plot

SPSS (Statistical Package for the Social Sciences) v11.5 for Windows was used to obtain the Receiver Operating Characteristic (ROC) plot for each of the SVM classifier developed in the study. The ROC curves were plotted using the scores obtained in the LOO cross-validation.

## Abbreviations

AAC: Amino Acid Composition; AUC: Area Under Curve; DPC: Dipeptide Composition; LOO: Leave-One-Out; MCC: Matthews Correlation Coefficient; PSI-BLAST: Position-Specific Iterative-Basic Local Alignment Search Tool; PSIPRED: Protein Structure Prediction; PSSM: Position-Specific Scoring Matrix; RBF: Radial Basis Function; ROC: Receiver Operating Characteristic; SCR: Structurally Conserved Region; SPSS: Statistical Package For the Social Sciences; SSC: Secondary Structure Composition; SVM: Support Vector Machine.

## Authors' contributions

JR carried out SVM experiments and developed the web server. JR and DG prepared the manuscript. DG supervised the project. Both the authors read and approved the final manuscript.

## Supplementary Material

Additional file 1 **Table showing the prediction accuracies with the 'other' kernels**. This is a complementary table to Table 1 and shows the prediction accuracies obtained with SVM models trained using kernels not shown in Table 1. This can be viewed using Microsoft Word. Click here for file

Additional file 2 **Positive dataset**. This consists of 136 sequences of full-length, annotated lipocalins used for training the SVMs. This can be viewed using any text editor like wordpad. Click here for file

Additional file 3 **Negative dataset**. This consists of 166 sequences of full-length, annotated non-lipocalins used for training the SVMs. This can be viewed using any text editor like wordpad. Click here for file

Additional file 4 **Blind test dataset for positives**. This consists of 42 full-length annotated lipocalin sequences not used in training or testing. This can be viewed using any text editor like wordpad. Click here for file

Additional file 5 **Blind test dataset for negatives**. This consists of 53 non-lipocalin sequences, comprising 25 FABPs and 28 triabins. This can be viewed using any text editor like wordpad. Click here for file
