# Supplementary material for: LipocalinPred: a SVM-based method for prediction of lipocalins
Source: BMC Bioinformatics. 2009 Dec 24;10:445. doi: 10.1186/1471-2105-10-445 (PMC2813246; doi:10.1186/1471-2105-10-445)
Supplement: Additional file 1 — Table showing the prediction accuracies with the 'other' kernels. This is a complementary table to Table 1 and shows the prediction accuracies obtained with SVM models trained using kernels not shown in Table 1. This can be viewed using Microsoft Word. [file 1471-2105-10-445-S1.DOC]

| Feature | Kernel | Parameters | | | | SN (%) | | SP (%) | Acc(%) | | MCC | | F measure | |
| --- | --- | --- | --- | --- | --- | --- | --- | --- | --- | --- | --- | --- | --- | --- |
|  | | Threshold | C | γ | d |  | | | | | | | | |
| AAC | L | -0.3 | 0.01 | - | - | 67.64 | 70.48 | | | 69.20 | | 0.380 | | 1.291 |
| AAC | P | -0.3 | 0.001 | - | 3 | 75.73 | 74.09 | | | 74.83 | | 0.496 | | 1.396 |
| DPC | L | -0.2 | 0.01 | - | - | 80.88 | 78.91 | | | 79.80 | | 0.595 | | 1.501 |
|  | R | -0.1 | - | 0.001 | - | 97.79 | 98.79 | | | 98.34 | | 0.966 | | 1.950 |
| PSSM | L | -0.1 | 38 | - | - | 84.55 | 86.14 | | | 85.43 | | 0.706 | | 1.649 |
| PSSM | P | -0.1 | 10 | - | 10 | 85.29 | 89.15 | | | 87.41 | | 0.745 | | 1.712 |
| SSC | L | -0.1 | 24 | - | - | 81.61 | 79.51 | | | 80.46 | | 0.608 | | 1.515 |
| SSC | P | 0.2 | 2 | - | 1 | 83.82 | 78.91 | | | 81.12 | | 0.624 | | 1.516 |
| DPC+SSC | L | 0.0 | 1 | - | - | 83.08 | 84.33 | | | 83.77 | | 0.673 | | 1.608 |
| DPC+SSC | R | 0.1 | - | 0.0001 | - | 83.08 | 87.34 | | | 85.43 | | 0.705 | | 1.669 |
| PSSM+SSC | L | 0.1 | 6 | - | - | 86.02 | 85.54 | | | 85.76 | | 0.713 | | 1.642 |
| PSSM+SSC | P | 0.1 | 5 | - | 1 | 84.55 | 84.33 | | | 84.43 | | 0.687 | | 1.614 |
| DPC+PSSM | L | -0.3 | 1 | - | - | 77.20 | 81.92 | | | 79.80 | | 0.591 | | 1.538 |
| DPC+PSSM | P | -0.4 | 0.01 | - | 3 | 79.41 | 82.53 | | | 81.12 | | 0.619 | | 1.560 |
| DPC+PSSM+SSC | L | -0.1 | 1 | - | - | 86.02 | 81.32 | | | 83.44 | | 0.670 | | 1.565 |
| DPC+PSSM+SSC | R | 0.1 | - | 0.0001 | - | 83.08 | 87.34 | | | 85.43 | | 0.705 | | 1.669 |
